# Implementing a peer-to-peer, self-management intervention for young people with depression and anxiety in Denmark

**DOI:** 10.1186/s40359-022-00777-w

**Published:** 2022-03-16

**Authors:** Sigurd Lauridsen, Maj Britt Dahl Nielsen, Amalie Oxholm Kusier, Camilla Øst Cloos, Marie Pil Jensen, Susan Andersen, Lau Casper Thygesen, Anna Paldam Folker

**Affiliations:** National Institute of Public Health, University of Southern Denmark, Studiestræde 6, 1455 Copenhagen, Denmark

**Keywords:** Anxiety and depression, Implementation evaluation, Community-based intervention, Self-management, Peer-support, Young people

## Abstract

**Background:**

Depressive and anxiety disorders share major risk factors and can often be effectively prevented or treated with similar interventions. However, less than half of young people with mental health problems seek professional help and hence innovative approaches to support this group are needed. To this end Coping with Anxiety and Depression shows promise. The aim of this paper was to evaluate the implementation of Coping with Anxiety and Depression for young people aged 15–25 years showing symptoms of anxiety and/or depression in a Danish community setting.

**Methods:**

The programme was implemented in 39 Danish municipalities. To evaluate the implementation of the programme, we collected quantitative and qualitative data simultaneously and subsequently we triangulated it in the data analysis. The qualitative data collection comprised semi-structured interviews in seven case municipalities. In each municipality we conducted interviews with a local municipality coordinator of the program, a group interview with two voluntary instructors facilitating the program and a group interview with two to seven participants in the programme. In total, seven local coordinators, 14 voluntary instructors and 23 participants (8 men and 15 women) were interviewed. We also carried out a baseline and post-intervention survey. Interview data was coded via an inductive and deductive analysis approach. Survey data was analysed via descriptive statistics in the statistical software programme STATA 16.

**Results:**

The evaluation showed that the implementation of the programme is feasible, and that most participants (83%) were satisfied with the programme to a high/very high extent. In total, 84% of the participants strongly agreed/agreed that it was advantageous that instructors themselves had experience with anxiety and depression. Qualitative data showed that the participants were very positive about the group format. It also showed that recruitment and retention of participants and especially young instructors were challenging but doable. Thus our findings suggest that the programme can be implemented in a way that is perceived as useful and satisfying to young people.

**Conclusions:**

Coping with Anxiety and Depression constitutes a feasible peer-to-peer, group-based community programme that is well-received by its target group. Recruitment and retention of participants and instructors is challenging but doable.

**Supplementary Information:**

The online version contains supplementary material available at 10.1186/s40359-022-00777-w.

## Background

Depressive and anxiety disorders are among the leading contributors to the health burden among children and young people globally [1–3]. The two disorders often co-occur and are often prevalent in youth [3]. Young people with depression are, for instance, six to twelve times more likely to have anxiety than are young people who are not depressed [4]. Moreover, depression and anxiety in adolescence predicts a range of mental health disorders in adult life, including substance-related disorders, bipolar disorder and suicidal behaviour as well as unemployment and physical health problems [5].

The World Health Organization therefore considers efforts to prevent these disorders a public health priority [6], and there is growing evidence that prevention programmes directed at children and young people can reduce symptoms and delay the onset of major anxiety and depressive disorders [7–9]. Notwithstanding, less than half of young people with mental health problems seek professional help [10–13]. Major barriers include lack of recognition of mental health problems and low mental health literacy, concerns about confidentiality and trust, difficulties navigating existing mental health services, and fear of judgement or stigma associated with mental illness and seeking professional help [11, 14–19]. Existing mental health services often provide delayed and restricted access to services for a small subgroup of young people with severe disorders with the artificial boundary at age 18 as a major barrier. In light of these barriers, innovative approaches supporting young people with mental health disorders are vital [20].

As anxiety and depressive disorders share major risk factors, they often can be effectively prevented or treated with similar interventions [21]. Interventions include school-based, community-based and digital-platform and individual-/family-based interventions [22, 23]. The two best studied psychological treatments are cognitive behavioural therapy and interpersonal psychotherapy, but to enhance reach there is an unmet need for other types of measures, preferably less expensive, low threshold, and community-based programmes [5, 24].

To this end, Coping with Anxiety and Depression, a peer-to-peer and group-based psycho-social programme aimed at improving self-efficacy and self-management among young people with symptoms of anxiety and depression, warrants interest. Originally, Coping with Anxiety and Depression was developed for adults (18 + years). An effect evaluation based on a randomized controlled trial showed that this programme increased self-efficacy and reduced symptoms of anxiety and depression among adults [25].

Coping with Anxiety and Depression for young people was initiated by the Danish Parliament, and implemented by the Danish Committee for Health Education, a non-profit NGO. It is focused on improving self-efficacy and self-management among young people with symptoms of anxiety and/or depression. Among other things, it stands out against other programmes in the following three respects: It is group-based, partially user-led (peer-to-peer) and community-based. This means that the programme is delivered in a non-clinical setting by non-clinical staff for a diverse group of young people ranging from the age of 15–25 with varying degrees of anxiety and depression and sometimes comorbid disorders. Moreover, the group format allows the inclusion of up to 14 participants at the same time, and may therefore be a cost-effective alternative to e.g., individual cognitive behavioural therapy and interpersonal psychotherapy—though the literature on the feasibility and cost-effectiveness of community-based programmes is sparse [26].

To deliver and disseminate high quality public health interventions it is central to understand implementation facilitators and barriers. That is, in regard to Coping with Anxiety and Depression for young people, to investigate e.g., whether this audience can be reached in a community setting and how can we refrain them from dropping out once enrolled in the programme. Process- and implementation research can help answer these questions and further our understanding of what, why, and how interventions work in “real world” settings [27].

### Aim

The aim of this paper is thus to evaluate the implementation of Coping with Anxiety and Depression for young people showing symptoms of, but not necessarily diagnosed with, anxiety and/or depression aged 15–25 years in a Danish community setting. More specifically the research question of this study was to assess barriers and facilitators to implementation of the programme (including dose delivered and received, recruitment and context).

## Methods

### Study design

To evaluate the implementation of the programme we employed both quantitative and qualitative methods. The study was designed as a QUAN + QUAL complementary study (simultaneous collection and analysis of quantitative and qualitative data, giving equal weight to both types of data) [28]. The qualitative data was used to provide in-depth understand of the implementation process, whereas the quantitative data was used to examine the magnitude and breadth of activities and perceptions. Data were collected through three surveys and qualitative interviews in seven selected case municipalities from the beginning of 2017 to the end of 2019.

This implementation study is part of a larger program evaluation aimed at assessing the program´s effectiveness on school and labour market attainment and changes in self-efficacy, depressive and anxiety symptoms measured before and after the intervention. The results of the effectiveness study will be presented in a forthcoming paper.

In the following we describe the content, structure, and inclusion criteria of the intervention program then the evaluation framework we used to investigate the implementation of the programme and finally the methods and strategies we used for collecting and analysing data.

### Content and structure of the intervention programme

The programme Coping with Anxiety and Depression is a peer-to-peer group-based programme consisting of seven 2.5-h modules deployed over seven weeks. The content of the programme is based on research literature on self-management and uses self-efficacy as a core outcome [29].

The programme was implemented in 39 Danish municipalities. The target group of the programme was young people aged 15–25. Each module covered 5–7 themes and activities, such as how to deal with complex emotions, cognitive techniques, positive thinking, communication, problem solving, use of action plans and cooperation with the healthcare system. The instructors who facilitated the programme used a highly structured manual. The instructors were trained to facilitate the process using themselves as positive role models. To do be able to do this, they were given 2 × 2 days of training. During training they were both educated in how to be an instructor and assessed in relation to whether they could perform this task. As part of their training, they received guidance on how to use themselves in a constructive way when interacting with participants. They were certified when completing their training. After 2 years, they receive 2–3 tutorials to be re-certified. This modus is repeated continuously every two years (see additional file 1).

The programme was organised in the following way in each municipality: There was a local coordinator from the administration in the municipality who recruited instructors to facilitate the programme and young people with depressions and/or anxiety to participate in the programme, two unpaid volunteering instructors, and up to 14 programme participants with depression and/or anxiety. The local coordinator was responsible for setting up the programme. This included conducting initial meetings with possible program participants, recruiting instructors, and advertising for and recruiting program participants. The Danish Committee for Health Education had designed advertisement materials the coordinator could use to recruit program participants. At the initial meeting, possible program participants were screened for eligibility using a screening manual developed by The Danish Committee for Health Education, the Danish Health Authorities and Danish experts on anxiety and depression. Modules were led by two instructors who both either had current or previous experiences with anxiety and/or depression. The programme is peer-to-peer based in a double sense: Because instructors themselves have or have had experience with anxiety and/or depression and because participants interact with and supported each other during the programme.

Instructors facilitated up to two implementations of the program.

In four of the municipalities, it was embedded in the municipalities´ health centre; in two municipalities the programme was embedded in the municipality’s labour market administration and in one case, it was embedded in the municipality´s community psychiatry.

### Inclusion criteria

Based on the following inclusion criteria, the coordinator in each municipality assessed eligibility in dialog with candidates, who themselves had approached the program:

- The person is between 15 and 25 years of age
- The person has self-assessed symptoms of anxiety and/or depression or currently has no self-rated symptoms of anxiety and/or depression, but the person has previous experiences with anxiety and/or depression.
- Anxiety and/or depression can be a secondary disorder to other illness
- The person has a desire to join a group and is motivated to learn how to use self-management techniques
- No severe cognitive challenges (the person can receive and process information at a level where it is considered that the person will benefit from the programme)
- The person participates voluntarily
- There is no evidence of aggressive behaviour

As part of the eligibility assessment, the coordinators used the Revised Children's Anxiety and Depression Scale (RCADS) to screen for anxiety and depression among young people aged 15–17. For people aged 18–25 the Major Depression Inventory (MDI) was used to screen for depression and the Anxiety Symptom Questionnaire (ASS) was used to screen for anxiety. The coordinators used the inclusion criteria as a guide as to whether young people should be offered to participate in the program. They also valued the conversation about the program with the young people highly. Whether the young people should participate in the program was decided together with the young people informed of how they met the inclusion criteria. The coordinators reported that the most important reasons for not participating included lack of motivation, being either below 15 years or above 25 years or having significant cognitive deficits.

### Evaluation framework

To evaluate the implementation of Coping with Anxiety and Depression, we have employed components of an evaluation framework designed by Saunders and colleagues [30]. We have employed the following components: Dose delivered, satisfaction (dose received), reach, recruitment, and context.

The implementation components used in this study are defined and applied as follows (see Table 1):

- Recruitment: The recruitment procedure of participants as well as instructors were evaluated by survey to the coordinators and interviews with coordinators and participants.
- Reach was defined as the proportion of participants who participated (participation rates) in the programme. This was evaluated by survey to the coordinators, a post-intervention survey to participants, and interviews with coordinators and participants.
- Dose delivered was defined as number of intended modules provided to participants. This information was achieved from the coordinators.
- Dose received/satisfaction was defined as the participants’ opinions and attitudes towards the modules. We used participant survey and interviewed participants, instructors, and coordinators to explore the participants’ experiences.
- Context was defined as factors and aspects of the environment which either hindered or facilitated the implementation. We used interviews with coordinators, instructors, and participants to describe the context surrounding the provision of modules and participation in them.

**Table 1:** Research themes and data collection

	Recruitment	Reach	Dose delivered	Dose received	Context
Quantitative data sources					
Survey, participants (baseline)		X			
Survey, participants (post-intervention)				X	
Survey, coordinators	X	X	X		
Qualitative data sources					
Interview, coordinators	X	X		X	X
Interview, participants	X	X		X	X
Interview, instructors				X	X

### Data collection

#### Qualitative interviews

The qualitative data collection comprised semi-structured interviews in seven case municipalities (each municipality is denoted: C1, C2 etc. throughout the paper). The interviews were conducted by two senior researchers (the first and second author) and a research assistant (the third author), all with extensive experience with qualitative interviews. Two of the interviewers had a background in public health and the third interviewer had a background in philosophy and psychology. In each municipality, the following interviews were conducted:

- an interview with the local municipality coordinator
- a group interview with two instructors
- a group interview with two to seven participants in the programme.

Case municipalities were selected to obtain maximal variation in geographical location, size of the municipality and embeddedness of the programme [31].

Interviews with instructors and participants were made face-to-face after completion of the final module in the programme. Coordinator interviews were either conducted over the phone or face-to-face. In three municipalities, the coordinator was also an instructor. In total, seven local coordinators, 14 instructors and 23 participants (8 men and 15 women) were interviewed. The interviewers did not have a relationship with the participants prior to the interviews, but the coordinators were familiar with two of the interviewers, because the coordinators also helped facilitate the quantitative evaluation. The interviewers explained the purpose of the evaluation and the interviews at the beginning of each interview. A separate semi-structured interview guide was developed for each of the three types of informants. The interview guide was inspired by the themes in the evaluation framework as outlined in Table 1.

#### Survey among participants

In cooperation with the local coordinators, we carried out a three-wave survey among the participants. The coordinators handed out a paper survey at the screening session (baseline) and at the last module (post intervention). Participants received an electronic survey if they were not present at the last module. All participants received an electronic survey five months after the first module (follow-up). This was the same follow-up timepoint as the study of the effect of the programme on adults. This paper only presents select survey data relevant for the implementation evaluation (data from the follow-up are not included).

In the introduction letter to the survey, we explained that it was voluntary to participate in the evaluation and that non-participation would not influence participation in the programme. We sent two reminders to participants who did not complete the surveys, either by email or text message.

The baseline survey assessed participants sociodemographic characteristics, drug use, depression (Becks’ Depression Inventory, BDI-II) and anxiety levels (Spielberger´s State-Trait Anxiety Inventory, STAI) and motivation and expectations for participation. The post-intervention survey included questions, we had devised to this study, about satisfaction with the programme and extent of drop-out and reasons for drop-out. The wording of the questions about satisfaction and absence is shown in Table 3. We used the same questions as a previous evaluation of the same programme for adults over 18 for comparison.

A total of 483 participants agreed to participate in the evaluation and completed the baseline survey. Among the baseline responders, 67% (n = 351) completed the post-intervention survey.

#### Survey among coordinators

After completion of the programme, we emailed an electronic survey to all coordinators (n = 39) via email. In total, 37 coordinators completed the survey. The survey encompassed questions about organisation, partners, recruitment, the screening session with possible participants, causes for drop-out and future implementation of the programme in the municipality.

### Data analysis

The data from the interviews were transcribed verbatim. All transcriptions were imported to the qualitative analysis software programme NVivo 12. They were coded via an inductive and deductive analysis approach, which means that the coding was guided by the interview guide and the research questions (deductive), but at the same time was open to new themes appearing in the material (inductive) [32]. The second and third author conducted the data analysis. Table 2 shows the overall themes, derived from our evaluation framework, and the more inductive subthemes emerging during data analysis. The final step of the analysis entailed integrating the results from the qualitative and quantitative analyse into a coherent analysis and narrative. We used the weaving approach that involve writing both qualitative and quantitative findings together on a theme-by-theme or concept-by-concept basis [33].

## Results

### Characteristics of participants and recruitment

In this section we present the characteristics of participants and the findings on recruitment of participants and instructors separately.

### Characteristics of participants

In total, 478 participated in the programme and in the evaluation. As shown in Table 2, most participants were women (72%) with a mean age of 20 years. About 24% were under 18 years and 55% were between 18 and 22 years. About half were still enrolled in education, while 8% were working and 36% were unemployed. Among those enrolled in education, 25% attended elementary school, 41% high school and 18% vocational training. At baseline, 17% had major depression and high anxiety; 5% minimal depression and anxiety and about 30% had not at any time received medical treatment for anxiety or depression. The following cut-off points were used to divide participants into these groups: Minimal depression (BDI-II < 14), Mild depression (> = 14 & < 20): Moderate depression (> = 20 & < 29): Severe depression (> = 29) [34]. Low anxiety (STAI <  = 39.5): Moderate anxiety (> 39.5 & <  = 59.5): High anxiety (> 59.5) [35] (see Table 3). Missing responses ranged from 0,2% (gender) to 4% (labour market status).

**Table 2:** Themes and sub-themes

Themes	Sub-themes
Recruitment	Recruitment of instructors
	Recruitment of participants
Reach	
Dose received	
Satisfaction	
Contextual barriers

**Table 3:** Characteristics of participants at baseline (n = 483)

Age, year, mean (SD)	20 (3)
Gender, n (%)	
Women	346 (72)
Men	136 (28)
Labour market status, n (%)	
Enrolled in education	257 (55)
Working	39 (8)
Unemployed	169 (36)
Type of education^a^, n (%)	
Elementary school	55 (25)
High school	91 (41)
Vocational training	40 (18)
Higher education	36 (16)
Cohabitation, n (%)	
Living alone	82 (17)
Living with family	276 (58)
Living with friends	10 (2.1)
Living with partner/spouse	78 (16)
Other	27 (5.7)
Support for managing anxiety and depression^b^, n (%)	
Psychiatric support	138 (29)
Medical treatment	147 (30)
Support from psychologist	233 (48)
Other	91 (19)
No support	92 (19)
Drug use, n (%)	
Yes	206 (44)
No	262 (56)
Depression score (BDI), mean (SD) (n = 401)	27 (11)
Anxiety score (STAI), mean (SD) (n = 436)	52 (12)
Symptom burden (BDI-II & STAI combined), n (%)	
Minimal depression AND low anxiety	24 (5)
Moderate anxiety (or lower) OR mild/moderate depression	148 (31)
Moderate anxiety AND moderate depression	73 (16)
Major depression OR high anxiety	145 (31)
Major depression AND high anxiety	80 (17)

^a^Among participants enrolled in education; ^b^The percentages add up to more than 100% because some participants receive more than one kind of support

### Recruitment of participants

We asked the coordinators about their experiences with recruitment of participants in a survey and qualitative interviews with coordinators and participants. In the survey, 41% (n = 16) of coordinators responded that recruiting participants was challenging to a very high degree/high degree, whereas 32% (n = 12) responded to some degree and 27% (n = 11) to a low/very low degree. In general, the coordinators in the qualitative interview study explained that it always takes time to create awareness about a new service. The interviews also showed that the coordinators used many different channels, such as local newspapers and Facebook, as well as general practitioner and local libraries. Most importantly, many coordinators established networks with professionals in other municipal departments, such as employment agencies, social services, and schools.

‘A lot of my work consists of getting out there and telling people that we are starting up a new thing here – and to try to figure out who the stakeholders are in this municipality and among non-for-profit organisations’ (Coordinator, C2).

Targeting recruitment efforts to other professionals seemed to be a productive strategy. Many participants explained that they heard of the programme via others, such as family members or professionals. This also concurs with the coordinator’s experiences. However, several participants highlighted that recruitment efforts could focus more on other social media, such as Instagram and Twitter:

‘I think that all young people are on Facebook, Instagram and Twitter […], a lot of young people think it is a taboo to ask for help. It´s not, but you don’t think that when you’re unwell, because you feel weak. But if it was more widely distributed via social media, then more young people would think that it is OK not to be OK’ (Participant, C3).

The interviews with the participants showed they had been motivated by different aspects of the programme. While some were attracted by the group format, others had been more attracted by the self-management concept. Several participants highlighted that the programme was free of charge and that they experienced a lack of service for people in their situation:

‘I felt like I missed that someone would take care of me and my problems after I turned 18. I didn’t really know where to start or who to turn to, so it was obviously a good opportunity, and it was free’ (Participant, C3).

### Recruitment of instructors

The peer-to-peer concept—a core element in the programme—entails that instructors must have first-hand experiences with symptoms of anxiety and/or depression. In this study, we also found that recruitment of instructors was a challenge, and about 54% (n = 21) of the coordinators responded that recruiting instructors was challenging to a very high/high degree; 19% (n = 7) responded to some degree and 27% (n = 11) to a low/very low degree. The coordinators in the qualitative study explained that finding instructors who had first-hand experiences with symptoms of anxiety and/or depression and, at the same time, had surplus energy to act as instructor, was particularly challenging.

‘It has been really, really hard to recruit instructors, because you need to have experience with anxiety and depression, but you also need to be very strong to be able to teach and have the surplus energy to show up every time, and many [instructors] have a job at the same time.’ (Coordinator, C4)

The recruitment of instructors was very essential because the programme could not run without instructors:

‘I think the most difficult part of the programme has been recruitment of instructors, because it is so vulnerable, it all depends on it.’ (Coordinator, C2)

The coordinators explained that they typically recruited instructors via external partners, such as volunteer organisations. Moreover, coordinators from municipalities which already offered the programme for adults over 18 years recruited instructors among the participants.

In an initial pilot test prior to this study, instructors also had to be the same age as the participants, but this demand was eventually withdrawn, because an evaluation of the pilot test showed that recruitment of instructors was particularly challenging and that the age of the instructors was not important for the participants.

Although the instructors did not have to be the same age as participants in this study, we found that several coordinators explained that they primarily looked for young instructors. However, because recruitment was often challenging, some coordinators chose to participate together with a volunteer peer-to-peer instructor or to mix a younger and an older instructor. In the section on satisfaction, we present the findings regarding the participants assessment of the instructors and the importance of age.

### Reach

In this section, we present participation rates. This project aimed to implement two programmes, with 12–14 participants in each programme, in 25 municipalities. In total, 39 municipalities participated; 29 successfully implemented both programmes, whereas 10 implemented one programme. During a first wave of implementing the programme, 44% of the coordinators reported that they enrolled 12 or more participants, but only 18% enrolled 12 or more participants during a second wave of implementation. The decrease in participants per programme reflected challenges with recruitment.

One third (31%) of participants reported that they attended all seven modules, whereas 57% reported to be absent between one and two times, and 12% three or more times (Table 2). Participants and coordinators in the qualitative study reported that tiredness and seediness was the main reason for absence. To increase participation, most local coordinators sent reminders and follow-ups by text message, phone call or email, and some coordinators arranged that someone would accompany the participants typically a parent or another relative. The participants responded favourably to these measures highlighting that it was nice ‘that someone was thinking of them’. In the quote below, one of the participants explains how the coordinator sent text messages prior to each module to encourage participation:

“´Bring your umbrella´ and things like that. It’s a cool message, because it gives a little spark, because you might not get out of the door when it’s raining” (Participant, C5).

#### Dose-delivered

Dose-delivered refers to the extent to which the programme was actual provided/delivered. All seven modules were executed as planned, i.e., dose-delivered was 100%.

### Satisfaction (dose received)

We assessed participants’ satisfaction with: (1) the screening session, (2) the programme, (3) the instructors and (4) the group format, respectively.

### Coordinators and participants´ satisfaction with the screening session

Before entering the programme, participants met with the coordinator to assess their eligibility. Both participants and coordinators felt that the screening session contributed to a sense of security, because the session gave the participants a better understanding of what to expect and prepared them emotionally for the programme.

‘Instead of just entering a room filled with all sorts of people that you never talked to be before, it was nice to talk to xxx (the coordinator) first. You never know exactly what to expect when you haven’t tried it before, but she could kind of explain what it was all about and what to expect. I really liked that’ (Participant, C4).

The manual for the coordinators stressed the importance of acknowledging participants’ viewpoints and to respect their perspectives. The interviews with the participants showed that participants in general felt that the coordinators received them in a positive and respectful manner.

‘I was afraid you had to argue why you need help, like in other parts of the system. Like, really convince them that you need to participate in this programme. But I didn’t experience that at all. It was more like, when I was there, I got the feeling that they just wanted to make sure that you came to the right place, and that they really wanted to help you’ (Participant, C2).

According to the coordinators, exclusion was always based on a mutual understanding and that the main reasons for exclusion was lack of motivation or severe cognitive challenges (as outlined in the section on content and structure of the programme).

‘It’s typically a mutual decision (…). I haven’t experienced that someone really wanted to participate and seemed really motivated, and I found myself saying no. But I have experienced that we, jointly, agreed that it was not right for them. Sometimes it simply comes down to that they are starting a lot of other things as well, for instance at school, at work or that they are starting some type of treatment or therapy, and that I could feel that they were a little bit stressed about all the things that were happening’ (Coordinator, C2).

While participants could bring a parent, social worker or friend to the screening session, coordinators generally had poor experiences with their presence and preferred that they waited outside, because they sometimes dominated the conversation too much.

‘Clearly it is different if they come alone or bring a parent. I think that the conversation is easier if they come alone, because the parents have a different perspective, and they come with expectations and hopes or sometimes they are more critical’ (Coordinator, C2).

### Satisfaction with the programme

About 83% of participants responded that they were very satisfied/satisfied with the programme, and 58% found the programme useful (to a very high/high degree), as shown in Table 4. In the qualitative study, the participants highlighted the relaxation exercises and action plans as particularly useful.

**Table 4:** Participation and satisfaction (post-intervention questionnaire)

	N	%
How many times were you absent from the seven course workshops?		
0	88	31
1–2	162	57
3 or more	35	12
The workshop leaders communicated the course material in an understandable and pedagogical manner		
Strongly agree/Agree	247	83
Neither agree nor disagree	17	6
Disagree/Strongly disagree	33	11
It was an advantage that the instructors had experience with anxiety and depression		
Strongly agree/Agree	243	84
Neither agree nor disagree	12	4
Disagree/Strongly disagree	35	12
Workshop leaders were skilled and well-prepared		
Strongly agree/Agree	246	82
Neither agree nor disagree	20	7
Disagree/Strongly disagree	34	11
To what degree was the course useful?		
Not at all/To some degree	126	42
To a high degree/To a very large degree	173	58
Altogether, how satisfied were you with the course?		
Very dissatisfied/Dissatisfied	10	3
Neither satisfied nor dissatisfied	42	14
Satisfied/Very satisfied	246	83
To what degree did you recognise the other participants’ problems and experiences?		
Not at all/A little	31	10
To some degree	69	23
A lot/To a large degree	201	67
To what degree did the other participants understand you?		
Not at all/A little	39	13
To some degree	65	22
A lot/To a large degree	195	65

Missing responses ranged from 7 to 9%, except the question about number of sessions which had 12% non-responses

The action plan was a self-management tool to help participants set goals and make plans for how and when to achieve these goals. Each week, the participants completed an action plan for the following week:

‘Those action plans have been really good, because you have something you can focus on, because when you have something to focus on and something to strive for, and when you have reached a milestone, then you feel that, well, it’s very cool, because you have actually achieved something, despite everything’ (Participant, C4).

A few of the participants felt that the action plans increased their beliefs in their ability to start an education; however, the coordinators and the instructors were generally more sceptical about the effect on education and labour market attainment, because most of the participants were already in school or at work.

While some of the participants found that the relaxation exercises were difficult, they generally felt that they were useful and something they could use after the programme as well:

‘I became quite fond of the relaxation exercises; I completely relax. In really think my body appreciates that it can just sink into a calm state and not be alert all the time. That’s something that I will take with me´ (Participant, C1).

#### Group dynamics

In the survey, 67% of participants reported that they could recognise the other participants’ problems and experiences, and 65% that the other participants understood them (a lot/to a large degree) (see Table 4). The participants in the qualitative study highlighted the social aspect of being part of a group of people with the same challenges. Thus, the group format allowed the participants to share their experiences. Although some participants were still in elementary schools and others were working and no longer lived with their parents, participants were able to relate in a meaningful way. Meeting others with a depression and anxiety induced a feeling of ‘being normal’, because many participants realised, they were not alone with their problems, which one participant explained as being able to ‘put a face on the disorder’. Although participants did not necessarily form friendships outside of the programme, they felt less lonely, because they realised that they were not alone with their problems.

‘It has been extremely good for me, because I don’t really know anyone with anxiety – besides this group. So, it’s very cool to find out that I am not abnormal; that I’m just a person with anxiety’ (Participant, C6)

#### The peer-to-peer concept and satisfaction with instructors

In the survey, 83% of the participants agreed that the instructors communicated the programme material in an understandable and pedagogical manner, and 82% that the instructors were skilled and well-prepared (agree/strongly agree). Furthermore, 84% of participants agreed that it was an advantage that the instructors had personal experience with anxiety and/or depression (agree/strongly agree). The qualitive interviews with instructors and participants revealed that the instructors functioned as role models, and that their first-hand experiences increased their credibility.

‘They [participants] clearly take example from us […] it’s not because we attended some fancy school that we say these things; it’s because we have actually lived with it. To be that kind of role model is a tool I use, and that experience is super. It means that they [participants] listen and that they trust us’ (Instructor, C6).

The participants had mixed feelings about the importance of the age of the instructors. Some preferred instructors who were about their age, whereas others preferred someone who was older, as exemplified in the two quotations below. Rather, the most important aspect of the peer-to-peer concept seemed to be personal experiences with anxiety and depression.

‘That they know our lives, I mean, for instance that they know that young people can feel a lot of pressure in school and all that stuff with social media and so on. Perhaps someone over 40 would not know about that the same way’ (Participants, C1).

‘I think it would be odd if it was someone our age, because there are people the same age – that’s the whole idea with the groups, so I think it’s OK that the instructors aren’t the same age as us, it makes good sense’ (Participant, C1).

## Contextual barriers and facilitators for implementation

We identified several contextual barriers and facilitators for the recruitment, retainment and satisfaction with the programme. We divided the facilitators and barriers into three main themes: *coordinator support*, *physical context,* and *organisational and geographical context*.

### The importance of Coordinator support for retainment and satisfaction

Some coordinators chose to stay in a room close to the programme venue to provide social support for participants and instructors. If any of the participants felt overwhelmed and left the programme, the coordinators were able to talk to them. The coordinators and instructors reported that the presence of the coordinator could prevent drop-out and provided participants and instructors with a sense of security.

‘I think it has ensured serenity and security that she [the coordinator] has been here. When some of the participants have dealt with anxiety, she has been able to meet with them instead of just leaving. I am quite sure our participants would have left, if it wasn’t for her’ (Instructor, C6).

### Pleasant physical area

The interviews with coordinators, instructors and participants showed that it was important to pay attention to the physical properties of the venue for the programme. In general, participants expressed satisfaction with the venues, which were easily accessible by public transportation or car. Moreover, ensuring that the venue had a roomy feel with sofas and serving snacks or food facilitated more informal interactions among the participants and contributed positively to the group.

‘We’ve made an extra effort to strengthen the sense of community. They get something to eat here, I mean, we try to make it cosy, and we have gone the extra mile, and the thing about eating together is nothing to sneeze at, because many of them live on their own and think it’s great to score dinner here. It sounds trivial, but I think … I mean it’s something you do together with someone who has the same challenges’ (Coordinator, C6).

In a similar vein, some of the participants explained how they experienced the atmosphere:

‘There’s always someone to greet you when you arrive and there’s always fruit available. So, you feel welcome.

Yes, they try to make it cosy, and the atmosphere is nice.

It doesn’t have a clinical feeling’ (Participants, C1).

The coordinators explained that they preferred that the programme was conducted in the same facility, because it felt safer for the participants. Moreover, this also allowed the coordinators to show the facilities to the participants at the screening session.

‘It means a lot that you can show them that this is where you are going to sit; you can choose where you want to sit because I will make a place card, so if they want to sit in the back at the right side of the room, then I will put the place card there’ (Coordinator, C6).

### The influence of organisational and geographical context on recruitment

The analysis shows that the organisational and geographical context seemed to have an impact on recruitment of participants. Firstly, the size of the municipality influenced the number of young people with symptoms of anxiety or depression and thus the size of the recruitment base. Secondly, the anchoring of the programme also played a role for the recruitment. While the majority (n = 25) of the municipalities anchored the programme in a health or health promotion department, others anchored the programme in an employment or education guidance department (n = 8), a social psychiatric unit (n = 5) or in a volunteer organisation (n = 1). It appeared to be a disadvantage to anchor the programme in a health and health promotion department because coordinators in these departments typically do not have direct access to and contact with young people. Consequently, they had to rely more on cooperating with external partners who did have such access than did coordinators in employment and education guidance departments.

## Discussion

In this study, we evaluated the implementation of Coping with Anxiety and Depression, a peer-to-peer group-based self-management programme for young people. The present study demonstrates that the programme is feasible in a community setting. We found that the programme was delivered as intended with high participation rates, although recruitment was challenging, especially regarding recruitment of the instructors. In total, about half of the local coordinators reported that recruiting participants into the programme was challenging to a high/very high degree, while one in four reported that recruiting participants into the programme was difficult to a high/very high degree. Such recruitment challenges are common, however. For instance, previous research shows that it is particularly challenging to recruit young men into mental health programmes, and that the stigma of mental health is a major barrier for recruitment of young people [36]. Likewise, in Coping with Anxiety and Depression, more women (72%) enrolled. However, this may also partly reflect that more women suffer from depression and anxiety. In Denmark, for instance, 8% of girls and 5% of boys up to 18 years of age are diagnosed with an anxiety disorder, and 2% of girls and 1% of boys up to 18 years of age are diagnosed with depression [37]. Moreover, recruitment challenges differed between municipalities, and findings point to different contextual factors that may promote or hinder recruitment. The size of the municipality appeared to be of importance. The programme’s recruitment base was simply larger in big compared to small municipalities, which made recruitment easier. The institutional anchoring of the programme also played a role. If the programme was implemented in an institution, the target group of young people already knew and regularly frequented this facilitated recruitment.

This study also showed that the programme was acceptable among young people. In total, 83% of the participants reported they were satisfied with the programme to a high/very high extent. The level of satisfaction is similar as among the adults in the previous intervention of the programme among adults (here, 71% reported that they were satisfied with the programme to a high/very high extent) [25]. Thus, while this paper has not addressed the effectiveness of Coping with Anxiety and Depression for young people, findings suggest that it can be implemented in a way that is perceived as useful and satisfying to young people.

The qualitative interviews showed that the participants especially liked the action plans and the relaxation exercises and that they were positive about the group format that allowed them to meet other people with similar challenges. In the survey, 67% reported that they recognised the other participants’ problems and experiences a lot/to a large degree and 65% reported that the other participants understood them a lot/to a large degree. This is important, since studies show that peer support increases participants’ sense of hope, control, and ability to effect changes in their lives as well as their self-care and sense of belonging to the community [38]. As children mature, settings and persons outside the family become increasingly important, and schools and peer exert a major influence. Young people are especially susceptible to peer influence about values, decision-making and the emotional displays of others. Hence, at this age, peer influence on health and well-being is greater than at any other age. Therefore, using peer-to-peer approaches in health interventions targeting young people may have immense potential [24].

In continuation of this, our survey showed that the majority of the participants reported they found it advantageous that instructors themselves had experience with anxiety and depression. The qualitative interviews supported this finding, but also revealed more mixed feelings about the importance of the age of the instructors. Informants reported that it was important that the instructors themselves had experienced anxiety or depression, while the age of the instructor was less important. Although peer support is a novel approach [39], it has been used in a variety of contexts, e.g., addiction and drug use and mental health services targeting different subgroups, such as families, young people, and people with disabilities [40–43]. The literature suggests that the benefit of peer support impact not only the recipient but also extends to peer support workers [44]. However, several challenges have also been identified, including low pay, stigma, unclear work roles, alienation, struggling with skill deficits, lack of training opportunities, emotional stress in helping others, and, on top of that, maintaining their personal physical and mental health. These barriers often result in high attrition rates among peer support workers in mental health settings[39]. Likewise, in our study we found that recruitment and attrition of instructors were challenging. Thus, to overcome this challenge some coordinators chose to mix instructors with experienced professionals, which may be a more pragmatic solution. Moreover, this study also highlights the importance of providing adequate support to instructors to ensure that they do not get overwhelmed.

This study thus indicates that Coping with Anxiety and Depression is a feasible community-based programme aimed at supporting young people with anxiety and/or depression. It would be relevant for future intervention research to investigate implementation of the programme in other cultural and organizational settings. The simplicity of the intervention suggests that transfer to other settings is possible (Additional file 1).

### Strength and weaknesses

It is a strength of our study that we have combined quantitative survey data from 39 municipalities (out of 98 municipalities) with qualitative in-depth data from seven case municipalities. This has enabled a thorough triangulated interpretation of the implementation of the programme and solid results on how Danish young people experience participating in Coping with Anxiety and Depression. It hereby contributes to the implementation research and knowledge on the implementation of mental health promotion programmes [26, 33]. It is, however, a limitation of the study that not all components of the methodological framework we used to analyse the implementation of the programme have been investigated with both quantitative and qualitative data. The context of the implementation has only been analysed qualitatively and not quantitatively. This was a methodological choice partly made because context can be difficult to capture in a survey and partly made to avoid a too lengthy survey. By avoiding too lengthy a survey we hoped to reduce the burden of the survey and hereby increase the response rate.

## Conclusion

To sum up, Coping with Anxiety and Depression constitutes a feasible peer-to-peer, group-based community programme that is well-received by its target group. The programme reaches its target group of young people with symptoms of anxiety and/or depression, and overall, the participants were satisfied with the programme. They can recognise the problems of the other participants and they appreciate that the instructors of the programme themselves have personal experience with anxiety and/or depression. Recruitment and retention of participants and instructors is challenging but doable. The results underscore the importance of partnerships across sectors, institutions, and departments, e.g., employment agencies, social services, and schools, as part of a successful recruitment strategy.

## Supplementary Information

**Additional file 1**. A description of the key elements in Coping with Anxiety and Depression

## Data Availability

The datasets used and analysed during the current study are available from the corresponding author on reasonable request.
