# Supplementary material for: Implementing a peer-to-peer, self-management intervention for young people with depression and anxiety in Denmark
Source: BMC Psychol. 2022 Mar 16;10:70. doi: 10.1186/s40359-022-00777-w (PMC8925241; doi:10.1186/s40359-022-00777-w)
Supplement: Supplementary file 1 — Additional file 1. A description of the key elements in Coping with Anxiety and Depression [file 40359_2022_777_MOESM1_ESM.docx]

A description of the key elements in Coping with Anxiety and Depression

Coping with Anxiety and Depression is a peer-to-peer and group-based psycho-social programme aimed at improving self-efficacy and self-management among young people (15 – 25) with symptoms of anxiety and depression.

The programme consists of seven modules and is facilitated by two certified voluntary instructors with personal experience with anxiety and/or depression. To become a certified instructor, a person must undergo 4 days of training in facilitating the programme.

Each of the 7 modules in the programme contains descriptions of the purpose of the module and of the module´s learning outcomes as well as a checklist of the materials that should be used to conduct the module. All modules consist of 5 to 7 activities and last approximately 2.5 hours. The programme is implemented over a period of 7 weeks.

To facilitate the modules instructors, receive a program manual which describes in detail the purpose and learning outcomes of the individual modules as well as how the activities in each module are to be carried out. Table 1 describes the activities of each module.

Table 1 Modules and activities

| **Modules** | **Activities** |
| --- | --- |
| **Module 1** | - Introduction to the course (20 minutes) - Group presentation by the participants (mention the mental illness or disorder you have + mention 1-2 problems you have because of your anxiety or depression (30 minutes) - Introduction to cognitive coping techniques (15 minutes) - Breathing techniques (15 minutes) - Introduction to the idea of an action plan (participants prepare a personal action plan) (30 minutes) - The instructors round off the day’s program (10 minutes) |
| **Module 2** | - Feedback/problem solving of the action plan from last module (30 minutes) - Dealing with difficult emotions (40 minutes) - Physical activity and mood (35 minutes) - Preparation of an action plan for the following week (20 minutes) - The instructor rounds off the day’s program (5 minutes) |
| **Module 3** | - Feedback on the action plan from last module (20 minutes) - Healthy food (20 minutes) - To make decisions (20 minutes) - Dealing with depression (20 minutes) - Positive thinking, with an exercise (25 minutes) - Preparation of an action plan for the following week (20 minutes) - The instructors round off the day’s program (5 minutes) |
| **Module 4** | - Feedback on the action plan from last module (20 minutes) - Killing myths, discussion (30 minutes) - Distraction, with an exercise (15 minutes) - Communication, reading and discussion (35 minutes) - Preparation of an action plan for the following week (15 minutes) - The instructors round off the day’s program (5 minutes) |
| **Module 5** | - Feedback on the action plan from last module (20 minutes) - Dealing with anxiety (40 minutes) - Triggers and warning signals of relapse (30 minutes) - Slackening, with an exercise (10 minutes) - Preparation of an action plan for the following week (15 minutes) - The instructors round off the day’s program (5 minutes) |
| **Module 6** | - Feedback on the action plan from last module (15 minutes) - Dealing with fatigue and sleep (25 minutes) - Fantasy journey, with an exercise (20 minutes) - To prevent anxiety and depression (25 minutes) - Proper use of medicine (25 minutes) - Preparation of an action plan for the following week (15 minutes) - The instructors round off the day’s program (5 minutes) |
| **Module 7** | - Feedback on the action plan from last module (20 minutes) - Collaboration with the healthcare system (25 minutes) - Flashback, discussion: what have we learned from the programme (35 minutes) - Goals for the future, a long-term action plan (35 minutes) - The instructors round off the whole program (10 minutes) |
